# The Impact of Ivermectin on Immune Response and Wound Healing in a Mouse Model Infected With *Leishmania major*

**DOI:** 10.1155/jotm/5579789

**Published:** 2025-08-17

**Authors:** Abdolbaset Rahmani, Nahid Maspi, Hamid Hasanpour, Shahab Falahi, Tooran Nayeri, Razi Naserifar

**Affiliations:** ^1^Department of Parasitology, School of Paramedicine, Ilam University of Medical Sciences, Ilam, Iran; ^2^Zoonotic Diseases Research Center, Ilam University of Medical Sciences, Ilam, Iran; ^3^Infectious and Tropical Diseases Research Center, Dezful University of Medical Sciences, Dezful, Iran

**Keywords:** immune response, ivermectin, Leishmania, transcription factors, wound healing

## Abstract

**Background:** Understanding the immune pathogenesis mechanisms of leishmaniasis is crucial for developing effective therapeutic interventions. In this study, we investigated the effect of ivermectin (IVE) on the expression of transcription factors GATA-binding protein 3 (GATA-3), T-bet, and ROR-γt as well as wound healing Leishmania infection.

**Methods:** Leishmania promastigotes were subcutaneously inoculated into the tail base of BALB/c mice (*n* = 10 per group) who later received phosphate-buffered saline (PBS), IVE, Glucantime, or a combination of IVE and Glucantime as soon as wounds developed, approximately three weeks' postinfection. The treatment continued daily for 2 weeks. The diameter of the wound was measured weekly over 6 weeks. In addition, in the sixth week, the mRNA expression of T-bet, GATA-3, and ROR-γt in splenic T cells was assessed through real-time PCR analysis.

**Results:** The findings of this research indicated a significant reduction in the lesion size among the treated groups compared with the control group (*p* < 0.05). IVE had a similar effect to Glucantime in reducing wound diameter (*p* > 0.05). Furthermore, the combined use of Glucantime and IVE led to the most reduction in the lesion size among all groups. The treated groups exhibited higher expression levels of T-bet and ROR-γt and lower levels of GATA-3 compared with the control group (*p* < 0.05).

**Conclusion:** IVE has shown significant efficacy in accelerating the healing process of wounds related to leishmaniasis. In addition, the administration of this medication has triggered a strong immune response, marked by activation of T helper 1 (Th1) and Th17 cells while also modulating Th2 responses.

## 1. Introduction

Leishmaniasis, caused by Leishmania protozoa, manifests in varying clinical forms and is influenced by host immune responses [1]. Protective immunity against Leishmania relies on T helper 1 (Th1)-driven responses (e.g., interleukin-12 and interferon-gamma) that activate macrophages to kill parasites via nitric oxide [2]. Conversely, T helper 2 (Th2) cytokines (e.g., interleukin-4 and interleukin-10) promote disease progression [3]. T helper 17 (Th17) cells also play a dual role in leishmaniasis. While Th17-associated cytokines like interleukin-17 can enhance neutrophil recruitment and inflammation, contributing to tissue damage, they may also support protective immunity in certain contexts by promoting Th1 responses [4, 5]. The differentiation of T helper subsets is governed by transcription factors T-box expressed in T cells (T-bet) for Th1, GATA-binding protein 3 (GATA-3) for Th2, and ROR-γt for Th17 [6–9]. The interaction of these factors in the differentiation of T helper cells determines the immune responses induced in the host infected with Leishmania and can lead to the improvement or progression of this disease [6].

Current therapies like pentavalent antimonials and amphotericin B face challenges including toxicity, resistance, and high costs [10–12]. Overall, the increasing resistance of infectious organisms to current medications, along with sporadic outbreaks of zoonotic, emerging, and reemerging infectious diseases, emphasizes the critical need for safer alternatives for treating leishmaniasis [13]. Ivermectin (IVE), a broad-spectrum antiparasitic, or its derivatives exhibits leishmanicidal activity against multiple Leishmania stages and has shown promise in managing cutaneous leishmaniasis [14–16]. Recent studies have also suggested that IVE has a positive impact on managing visceral, tegumentary, and cutaneous forms of leishmaniasis [15–18]. However, its immunomodulatory effects on T-cell transcription factors and wound healing remain unexplored. This study aimed to determine the effect of IVE on the expression of transcription factors T-bet, GATA-3, and ROR-γt, as well as its influence on wound healing in mice infected with *Leishmania major*.

## 2. Methods

### 2.1. Multiplication of Leishmania Promastigotes

The Iranian strain of *L. major* promastigotes (MHROM/IR/75/ER) was obtained from the Faculty of Health at Tehran University of Medical Sciences. They were cultured in RPMI 1640 medium (Gibco) containing penicillin (100 IU/mL) and streptomycin (100 g/mL), enriched with 15% heat-inactivated fetal bovine serum (FBS), at a temperature of 24°C.

### 2.2. Study Animals

In this study, 40 female BALB/c mice aged 4–6 weeks were obtained from the laboratory animal breeding department of the Pasteur Institute of Iran in Tehran. The mice were housed in optimal environmental conditions, maintained at a temperature of 20°C–22°C, with approximately 50% humidity, and were subjected to a light cycle of 12 h of light followed by 12 h of darkness. After 1 week of acclimatization, the mice were used for experiments.

### 2.3. Animal Inoculation and Induction of Leishmanial Lesions

Leishmania promastigotes in the stationary phase were used to induce leishmanial lesions. Two million parasites suspended in 100 μL of phosphate-buffered saline (PBS) were injected subcutaneously into the base of each mouse's tail. After infection, the mice were randomly divided into four groups (*N* = 10 mice per group): a negative control group receiving PBS, a group treated with Glucantime at a dose of 20 mg/kg/day via intramuscular injection, a group given IVE at 200 μg/kg/day, through the intraperitoneal route, and a final group administering both IVE and Glucantime simultaneously. After 3 weeks, during which wounds developed at the base of the tails, drug therapy was initiated and continued daily for 2 weeks. The wound diameters were measured weekly using a caliper.

### 2.4. Assessment of mRNA Expression Levels for Transcription Factors

Six weeks after the initiation of treatment, an investigation was conducted to analyze the gene expression patterns of specific transcription factors in splenic cells including T-bet, GATA-3, and RORγt are lineage-defining transcription factors for Th1, Th2, and Th17 cells, respectively. Their mRNA expression in spleen tissue directly reflects T-cell activity, as validated in prior studies [6–9].

To facilitate this study, the mice were euthanized, and their spleens were carefully isolated under aseptic conditions. The SYBR Green real-time (RT)-PCR technique was used to measure mRNA expression levels of transcription factors in T cells from spleen tissues. Total RNA was extracted directly from homogenized whole spleen tissue using the RNJia RNA kit (Roje Technologies, Iran). Spleens were mechanically homogenized, followed by RNA extraction using the RNJia RNA kit. Finally, the RNA was washed and dissolved in RNase-free water. DNase treatment was applied to remove genomic DNA.

Experiments were repeated three times (biological replicates, *n* = five mice/group/replicate). RNA was extracted from at least three independent batches of spleen samples. RNA quality was assessed by A260/A280 ratios: 1.8–2.0 (measured via spectrophotometry, NanoDrop); also, RNA integrity was confirmed by agarose gel electrophoresis (sharp 18S/28S ribosomal bands).

Complementary DNA (cDNA) was then generated from 50 μg of total RNA samples following the instructions of the First Strand cDNA Synthesis kit (Yekta Tajhiz Azma Co, Tehran, Iran). The mRNA expression levels of T-bet, GATA-3, and ROR-γt transcription factors were examined using specific primers outlined in Table 1 and the RT-PCR technique using RealQ Plus 2 × Master Mix Green (Ampliqon, Denmark) and a LineGene K RT-PCR Detection System (Bioer Technology, Hangzhou, China). This method efficiently captures T-cell-specific gene expression without needing cell sorting. Melting curve analysis was conducted on all final PCR products to confirm amplification specificity. The efficiency of RT-PCR and relative quantification of gene expression were analyzed using the comparative 2 – ΔΔCt method [23]. All gene expression levels were normalized using the β-actin gene as an internal control.

### 2.5. Statistical Analysis

The collected data were analyzed using GraphPad Prism software (GraphPad Software Inc., La Jolla, CA, USA, Version 8.3). The normality of the data was assessed using the Kolmogorov–Smirnov test. To evaluate the differences in mRNA expression levels of transcription factors among the various groups, a one-way analysis of variance (ANOVA) was conducted, followed by post hoc Tukey multiple comparison tests. Furthermore, to examine the changes in average wound diameter across different groups over a 6-week posttreatment period, a two-way repeated-measures ANOVA with post hoc Tukey tests was utilized. A significance level of *p* < 0.05 was set for the analysis.

## 3. Results

### 3.1. Healing of Leishmania Lesions

**Figure 1:**
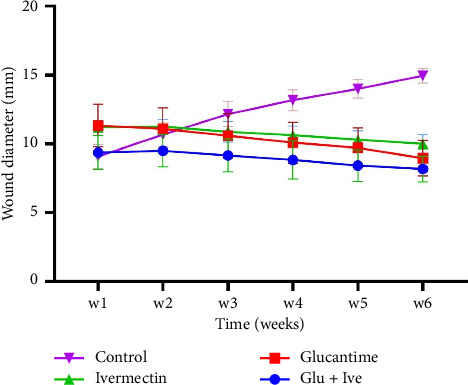
Average lesion size in mice infected with *Leishmania major* was measured for 6 weeks after the beginning of the treatment. “Glu + Ive” is an abbreviation for Glucantime + ivermectin (the raw data for Figure 1 (lesion size measurements) are provided as Supporting Table S1 at the end of the manuscript).

The progression of lesion size in *L. major*-infected mice was monitored weekly for 6 weeks posttreatment initiation. As detailed in Supporting Table 1, all treatment groups showed significant lesion reduction compared with untreated controls (*p* < 0.0001). A pronounced reduction started from the fourth week in the medicated groups, while the control group exhibited progressive lesion enlargement. No statistically significant difference in the mean lesion size was observed between the groups treated with IVE alone and Glucantime alone (*p* > 0.05). Similarly, the combination of Glucantime and IVE did not yield a significant difference in wound diameter compared with Glucantime monotherapy (*p* > 0.05). In contrast, the combination therapy group showed a notable reduction in the mean lesion size compared with the IVE-only group (*p*=0.04) (Figure 1). By the sixth week posttreatment, the wound healing rates relative to the control group were 32.12% for IVE, 40.14% for Glucantime, and 45.42% for the combination therapy.

### 3.2. mRNA Expression Levels for Transcription Factors

#### 3.2.1. T-bet Gene Expression

**Figure 2:**
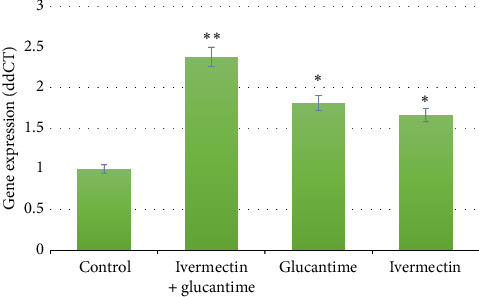
The T-bet gene expression levels were measured in mice infected with *Leishmania major* 6 weeks after treatment. Statistical significance was denoted by ^∗^ for *p* < 0.05 and ^∗∗^ for *p* < 0.01.

The results of the RT-PCR analysis indicated a significant overexpression in the groups that received IVE, Glucantime, or a combination of IVE and Glucantime, as compared with the control group (*p* < 0.05). There was no statistically significant difference in the mRNA expression levels of the T-bet gene between the groups treated with IVE and Glucantime (*p* > 0.05). Th1 activation was demonstrated by significantly elevated T-bet expression in all treated groups compared with the control group (*p* < 0.01; Figure 2).

#### 3.2.2. GATA-3 Gene Expression

**Figure 3:**
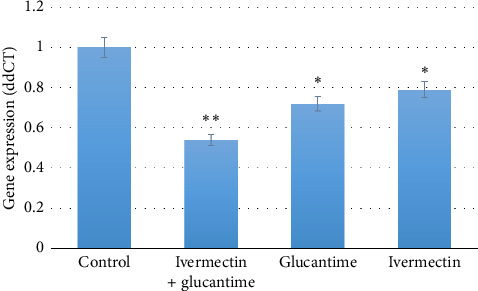
The GATA-3 gene expression levels were measured in mice infected with *Leishmania major* 6 weeks after treatment. Statistical significance was denoted by ^∗^ for *p* < 0.05 and ^∗∗^ for *p* < 0.01.

GATA-3 (Th2 transcription factor) expression was significantly decreased in all treatment groups versus control (*p* < 0.05), with maximal suppression observed in the IVE + Glucantime group. No significant difference existed between IVE and Glucantime monotherapies (*p* > 0.05; Figure 3).

#### 3.2.3. ROR-γt Gene Expression

**Figure 4:**
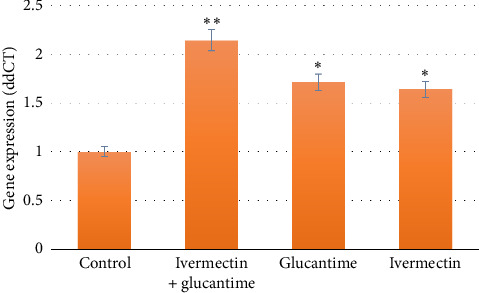
ROR-γt gene expression in spleen T cells of *Leishmania major*-infected mice quantified using real-time PCR within sixth week posttreatment (^∗^*p* < 0.05 and ^∗∗^*p* < 0.01).

RORγt (Th17 transcription factor) expression was significantly increased in all treated groups versus control (*p* < 0.05), peaking in the IVE + Glucantime group, with no significant difference between IVE and Glucantime monotherapies (*p* > 0.05; Figure 4).

## 4. Discussion

Neglected tropical diseases (NTDs) encompass a variety of diverse infectious diseases that are more prevalent in tropical and subtropical regions. These diseases are chiefly found in impoverished areas with limited access to healthcare facilities. Leishmaniasis is one such infectious NTD caused by a protozoan parasite of the genus Leishmania, transmitted to humans through the bite of an infected sandfly [16]. In Iran, pentavalent antimony compounds are the most commonly used drugs for treating CL. However, there have been reports of drug resistance emerging in *L. tropica* and *L. major* [24, 25].

In the present study, the antileishmanial effect of IVE on the expression levels of transcription factors of T cells and wound healing was evaluated in a mouse model of *L. major* infection. In all treated mice, leishmanial lesions gradually decreased over time compared with the control group. The group that received both drugs simultaneously showed most significant reduction in wound diameter, with a 45% decrease in the mean lesion size compared with the control group. In addition, Glucantime, used as the reference drug, displayed a similar effect to IVE in inducing wound healing. Our findings support previous studies by Cairns et al., which emphasized the role of IVE in increasing wound healing by assisting in the regeneration of peripheral nerves [26]. Another investigation revealed that the application of IVE cream on the skin facilitated wound healing and reduced several macroscopic indicators of wounds, including exudation, edge edema, hyperemia, and the accumulation of granulation tissue. This beneficial effect was achieved through multiple mechanisms, such as the modulation of the inflammatory response, as well as the regulation of vascular endothelial growth factor and transforming growth factor-beta 1 levels [27]. IVE may contribute to the healing of leishmanial lesions through one of the mechanisms previously discussed, making it a viable alternative treatment in regions where Glucantime is either unavailable or where resistance to this medication has developed. Nonetheless, additional research is essential to thoroughly investigate this possibility. Our findings are also in agreement with a study conducted by Kadir et al. In a comparative study, they demonstrated the antileishmanial effects of IVE against *L. tropica* in both in vitro and in vivo settings. This drug resulted in the most significant reduction in viable promastigotes compared with other medications such as rifampicin, Amp B, and nystatin. In addition, when 200 μg/kg of IVE was administered subcutaneously to mice infected with *L. tropica* for 5 days, there was a significant reduction in the mean lesion scores (MLSs). Ultimately, the lesions completely healed, reaching a score of zero within 1 month after treatment. On the other hand, mice treated with topical IVE had an MLS of 3 and a cure rate of only 40%. The researchers concluded that the variations in antileishmanial effectiveness between the various administration routes may be attributed to the inadequate drug absorption through the skin and its limited ability to effectively target intracellular amastigotes [14]. Furthermore, Rasheid and Morsy revealed that *L. major* promastigotes when pretreated with a single dose of 100 μg of IVE were unable to develop skin lesions at the inoculation site of Syrian golden hamsters, whereas the parasite was still isolated from their spleens and livers. Promastigotes either died or lost their infectivity after being treated for 2 days with 90 μg/mL [28]. As a result, our findings align with those found in the literature and strongly recommend the use of IVE as a safer treatment option for cutaneous leishmaniasis.

In this study, RNA was extracted directly from homogenized whole spleen tissue, not from isolated T-cells. While T-bet, GATA-3, and RORγt are T-cell-specific transcription factors, their mRNA expression in whole spleen tissue reflects the global transcriptional activity of splenic T cells, as the spleen is a primary lymphoid organ rich in T lymphocytes. This approach is well established in immunology studies for assessing T-cell responses [6–9]. The analysis of gene expression levels related to transcription factors in the treated animals when compared with the control groups indicated a predominance of expression of T-bet and ROR-γt genes and low expression of the GATA-3 gene compared with the control group. IVE or IVE + Glucantime combination treatments significantly altered T-helper cell transcription factor profiles, indicating a Th1/Th17-polarized response with concurrent Th2 suppression. This shift is unequivocally supported by RT-PCR gene expression analysis: Th1 activation was demonstrated by significantly elevated T-bet expression in all treated groups compared with the control group. Similarly, Th17 activation was evidenced by significantly increased RORγt expression in all treated groups, with expression peaking in the IVE + Glucantime group. Conversely, Th2 modulation was shown by significantly reduced GATA-3 expression in all treated groups, with maximal suppression observed in the IVE + Glucantime group. Critically, for all three genes (T-bet, GATA-3, and RORγt), mRNA expression levels did not differ significantly between groups treated with IVE alone versus Glucantime alone (*p* > 0.05). The findings indicate that the administration of IVE elicited immune responses characterized by the activation of Th1 and Th17 pathways, while simultaneously suppressing the Th2 response in mice infected with *L. major*. These observations corroborate earlier studies that reported the development of protective immune responses following IVE treatment. For instance, IVE has been shown to exert considerable antileishmanial activity against *L. infantum* by diminishing the population of amastigotes within treated macrophages. Furthermore, the treatment of BALB/c mice infected with *L. infantum* using either IVE or its encapsulated formulation in polymeric micelles has been associated with the enhancement of a protective Th1 immune response and a reduction in the parasitic load within their tissues [16]. In another study, it was also demonstrated that IVE and some of its synthetic derivatives have leishmanicidal activities against promastigote and amastigote forms of *L. amazonensis*, although their mechanism of action was not evaluated [15]. Clinical improvement manifests by Week 4 posttreatment, underscoring the delayed onset of anti-Leishmania action of the drugs. While our whole-tissue approach captures global T-cell responses, future studies with sorted T-cells could provide finer mechanistic insights.

## 5. Conclusion

Our findings revealed that IVE plays a role in curing leishmanial wounds caused by *L. major*. The control group exhibited progressive lesion enlargement throughout the study period, indicating no spontaneous resolution. A significant reduction in lesion diameter was observed in the treated groups relative to the control group. Marked improvement was initiated from the fourth week across the treatment groups. Combination therapy (Glu + IVE) demonstrated a 45.42% reduction in the lesion size compared with the control group that was the most pronounced lesion regression relative to all groups. Moreover, an increase in the expression of T-bet and ROR-γt genes and a decrease in GATA-3 expression occurred after administering IVE. This interaction of transcription factors indicates that the stimulation of inflammatory immunity and the regulation of anti-inflammatory immunity have occurred, eventually leading to the differentiation of cellular immunity, parasite death, and wound healing. One of the mechanisms of action of IVE in treating leishmaniasis is the change in the expression of transcription factors of T-cells. Moreover, IVE and Glucantime had synergistic effects on the expression of Th1 and Th17 transcription factors and could cause further improvement of the disease. In conclusion, our findings suggest that IVE can likely serve as an alternative drug to pentavalent antimonial compounds in regions experiencing resistance, and limited access to Glucantime, and as a safer treatment option against CL. While our whole-tissue approach captures global T-cell responses, future studies with sorted T-cells could provide finer mechanistic insights.

## Figures and Tables

**Table 1:** Primer sequences of transcription factor genes used in the real-time PCR technique.

Genes	Primers	Nucleotide size	Primer sequence (5′ ⟶ 3′)	References
β-Actin	Forward	20	CAGCCTTCCTTCTTGGGTAT	[19]
	Reverse	20	TGGCATAGAGGTCTTTACGG	

ROR-γt	Forward	20	TGCAAGACTCATCGACAAGG	[20]
	Reverse	20	AGGGGATTCAACATCAGTGC	

T-bet	Forward	20	GCCAGGGAACCGCTTATATG	[21]
	Reverse	23	GACGATCATCTGGGTCAGATTGT	

GATA-3	Forward	25	GAGGTGGACGTACTTTTTAACATCG	[22]
	Reverse	18	GGCATACCTGGCTCCCGT	

## Data Availability

The data that support the findings of this study are available on request from the corresponding authors.

## Supporting Information

Supporting Table 1. Mean wound diameters (mm, mean ± SD) in *Leishmania major*-infected BALB/c mice (*n* = 5 mice/group) over 6-week posttherapeutic intervention.
